# A gemini surfactant-containing system with abundant self-assembly morphology and rheological behaviors tunable by photoinduction†

**DOI:** 10.1039/c8ra01070f

**Published:** 2018-04-30

**Authors:** Yan Tu, Mengge Gao, Hongni Teng, Yazhuo Shang, Bo Fang, Honglai Liu

**Affiliations:** Key Laboratory for Advanced Materials, School of Chemistry & Molecular Engineering, East China University of Science and Technology Shanghai 200237 China shangyazhuo@ecust.edu.cn; Department of Applied Chemistry, College of Chemical and Environmental Engineering, Shandong University of Science and Technology Qingdao 266510 China; Shanghai Key Laboratory of Multiphase Materials Chemical Engineering, Lab of Chemical Engineering Rheology, East China University of Science and Technology Shanghai 200237 China fangbo@ecust.edu.cn

## Abstract

Photoresponsive micellar systems with adjustable aggregate morphologies and rheological properties may be useful in a number of fields such as in microfluidics, controlled release, and sensors. However, the complexity and great difficulty of synthesising photosensitive molecules hamper their practical applications to a significant degree. In this study, we constructed a novel photoinduced self-assembly system by introducing the photoresponsive derivative *trans*-2-methoxy-cinnamate (*trans*-OMCA) into the gemini surfactant *N*,*N*′-bis(dodecyldimethyl)-1,2-ethane diammonium dibromide (12-2-12·2Br^−^) solutions. The system displays abundant phase behaviors, and the long worm-like micelles, vesicles, as well as an aqueous two-phase system (ATPS) are observed in the 12-2-12·2Br^−^/*trans*-OMCA mixed system even at lower surfactant concentrations. The UV-responsive behavior of the formed vesicles and the worm-like micelles is investigated systematically. The results have shown that OMCA undergoes photoisomerization from the *trans*-form to the *cis*-form through UV light irradiation that alters the molecular packing at the micellar interface and thus leads to the transformation of micellar morphologies. The long worm-like micelles will turn into much shorter units when the sample is exposed to 365 nm UV light accompanied by a decrease in solution viscosity by more than an order of magnitude. The formed vesicle system, however, can be utilized to generate a multi-state self-assembly structure, including a worm-like micelle and a small spherical micelle, depending on the UV irradiation time. The morphologies of micelles in a 12-2-12·2Br^−^/*trans*-OMCA mixed system can be tailored by adjusting the system composition and the duration of UV light irradiation. Correspondingly, the rheological behavior of the 12-2-12·2Br^−^/*trans*-OMCA mixed system can be purposely tuned. The light-induced system with abundant self-assembly behaviors and tunable rheological properties would widen the potential application of gemini surfactants in drug delivery, smart fluids, and materials science.

## Abstract 2

The photoisomerization of OMCA affects the degree of OMCA participation in the formation of mixed micelles and results in the transformation of micellar morphologies.

## Graphical abstract

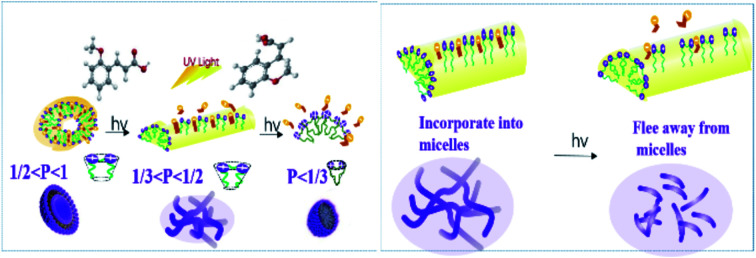

## 1 Introduction

Surfactants can self-assemble into various ordered structures including spherical micelles,^1^ vesicles,^2,3^ rod or worm-like micelles,^4–7^ as well as liquid crystals.^8,9^ These surfactant assemblies with various morphologies have attracted widespread attention because of their wide application in colloids, templates, and soft matter materials.^10,11^ Vesicles are very important in biomedicine as good candidates for drug delivery and biomimetic membranes.^12,13^ Worm-like micelles (WLMs)^14^ composed of long flexible aggregates of surfactant molecules in solution may tangle together into a transient three-dimensional network and display unique rheological behavior. WLMs have been applied in many fields such as in cosmetic products,^15^ oil recovery,^16^ rheology control,^17^ heat transfer,^18^ and drag reduction.^19^

Recently, stimuli-responsive smart micelles have attracted significant attention and interest from worldwide researchers due to their potential applications such as in controlled release of drugs, chemical separation, smart fluids, and sensors. These external stimuli include light,^20–22^ electricity and magnetism,^23^ CO_2_,^24,25^ pH,^26^ temperature,^27^ and redox reactions.^28^ Among these external stimuli, light is expected to be more advantageous and of significant importance.^29–31^ At first, light does not intervene in the composition of the system. In addition, it is a kind of clean energy and readily available. Moreover, photostimulation can be accurately localized, which is quite important especially in nanoscience. Finally, parameters such as wavelength and illumination time can be easily regulated to manipulate the structure and rheological properties of the systems. Therefore, over the past few decades, a large number of researchers have reported the self-assembly of surfactants in solution triggered by light. Generally, the main way of forming an optical response self-assembly system is to introduce photoresponsive groups into surfactant molecules. For example, Zhao *et al.* synthesized a carboxylate gemini surfactant, *O*,*O*′-bis (sodium-2-tetradecylcarboxylate)-*p*-azodiphendiol (G14-azo), constructed an equally charged mixture with the cationic surfactant cetyltrimethylammonium bromide (CTAB) in cyclohexane to form reverse worm-like micelles, and realized a transition of aggregate morphology from reverse worms to simple reverse micelles under UV-light irradiation.^32^ Wang *et al.* synthesized a cationic azobenzene surfactant, 4-cholesterocarbonyl-4′-(*N*,*N*,*N*-triethylamine butyloxyl bromide) azobenzene (CAB), and then mixed CAB and SDS together to form a photo-sensitive catanionic self-assembly system for drug delivery.^33^ To date, a variety of photoresponsive surfactants have been synthesized and used to induce light-responsive self-assembly of systems.^34–36^ However, the synthesis of the photoresponsive surfactants is a time-consuming, energy-consuming, and complicated work, which limits the development of light responsive self-assembly systems to some degree. Consequently, another simple and feasible way to construct a light-responsive self-assembly is developed that involves the addition of light-sensitive molecules to surfactant solutions directly.^37–40^ During the past few decades, significant efforts have been devoted to studying the photoresponsive behavior of a mixed system containing surfactants and light-sensitive molecules. Huang *et al.* established photo-modulated multistate molecular self-assemblies by utilizing cetyltrimethylammonium bromide (CTAB) mixed with sodium (4-(phenylazo) phenoxy)-acetate (AzoNa).^40^ Yu *et al.* used imidazolium-based SAIL and sodium azobenzene 4-carboxylate (AzoCOONa) to construct viscoelastic worm-like micelles.^37^ It is worth mentioning that cinnamic acid and its derivatives are also a class of commonly used photoresponsive compounds and have been paid significant attention due to their wide application in food, medicine, daily chemistry, and so on.^41^ Raghavan *et al.* developed a simple class of photorheological fluid using CTAB and *trans-ortho*-methoxycinnamic acid (OMCA) and realized the viscosity tunable by the photoisomerization of *trans*-OMCA to *cis*-OMCA under UV irradiation.^42^ The phase transition from bilayer vesicles to worm-like micelles for the photoresponsive aqueous system composed of tetradecyldimethylamine oxide and *para*-coumaric acid can also be explored systematically.^43^ Zheng *et al.* investigated worm-like micelles in a photo-modulated self-assembly system of the surface active ionic liquid C_16_mimBr/*trans*-cinnamic acid (*trans*-CA).^44^ Recently, Dai's group and Li's group also carried out a great deal of work and made significant achievements in this area.^38,39,45,46^ The properties of both the light-sensitive molecules and surfactants in the mixed system play essential roles in determining the photoresponsive behaviour of the system. The length and the structure of surfactant alkyl chains, head groups, as well as charges carried by the surfactant head groups affect the photoresponsive behavior of the mixed system directly.^47^

Gemini surfactants are famous not only for their structural diversity upon variation in the chemical structure of head groups, length and structure of alkyl chains, spacer configuration, and the counter-ions,^50^ but also their unique physicochemical properties including much lower critical micelle concentration and richer aggregation behaviors as compared to their conventional counterparts bearing a single alkyl chain and single head group.^48–51^ The structural diversity and rich aggregation behavior of gemini surfactants provide a premise for the construction of novel photoresponsive systems. However, to the best of our knowledge, the number of studies reported on photoresponsive systems containing gemini surfactants is rather less;^52^ further study on the photoresponsive behavior of a mixed system containing gemini surfactants is definitely worthwhile.

In this study, we combined the gemini surfactant *N*,*N*′-bis(dodecyldimethyl)-1,2-ethane diammonium dibromide (12-2-12·2Br^−^) and photoresponsive molecular *trans*-2-methoxycinnamic acid (*trans*-OMCA) for constructing a novel photoresponsive system. The phase behavior of the 12-2-12·2Br^−^/*trans*-OMCA system and the UV-responsive behavior of the formed vesicles and the worm-like micelles were investigated systematically. A possible mechanism for the aggregation behavior before and after irradiation for the 12-2-12·2Br^−^/*trans*-OMCA system is proposed. The results are supported by rheology measurements and TEM observation. We expect that the present study on the light-induced system with abundant self-assembly behaviors and tunable rheological behaviors would be of theoretical and practical importance.

## 2 Experimental

### 2.1 Materials

**Scheme 1:**
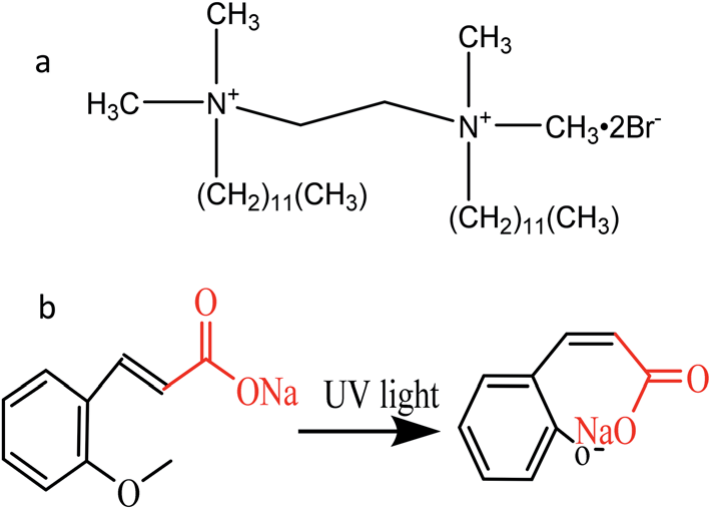
The chemical structure of *N*,*N*′-bis (dodecyldimethyl)-1,2-ethane diammonium dibromide (12-2-12·2Br^−^, a) and *trans*-2-methoxycinnamic acid (*trans*-OMCA, b).

*N*,*N*-Bis(dodecyldimethyl)-1,2-ethane diammonium dibromide (12-2-12·2Br^−^) was synthesized according to our previous study.^53^ The structure of 12-2-12·2Br^−^ was ascertained by ^1^H NMR spectroscopy using Bruker Avance 400. The spectrometer elemental analysis (Vario EL III) showed that the purity of the synthesized 12-2-12·2Br^−^ was above 97%. *Trans*-2-methoxycinnamic acid (99%) was purchased from TCI Chemical Industry Development Co., Ltd and used without further purification. Moreover, *trans*-2-methoxy cinnamate (*trans*-OMCA) used in this study was prepared by *trans*-2-methoxycinnamic acid with a slight excess of sodium hydroxide (NaOH) in solution. Their structural diagrams are shown in Scheme 1. Ultrapure water with a resistivity of 18.2 MΩ cm obtained using the Millipore system was used for all experiments.

### 2.2 Sample preparation

Gemini surfactant (12-2-12·2Br^−^) was dissolved in ultrapure water to form stock solutions with different concentrations. A series of 12-2-12·2Br^−^/*trans*-OMCA mixed solutions with the desired compositions was prepared by quantitatively adding *trans*-OMCA to the stock solutions, followed by standing in an incubator (25 °C) for two days to ensure equilibrium.

### 2.3 Phase diagram determination

Approximately 400 samples, each 3–5 g, were prepared to cover a wide range of compositions in the ternary phase diagram. The boundaries between different phase regions are assigned by visual appearance with an accuracy of better than 2%. Some representative samples from each region are selected for microstructure determination; these samples are primarily along the phase boundaries.

### 2.4 UV-light irradiation

An ultra-high pressure short arc mercury lamp (CHF-XM35-500W) with a 365 nm optical filter was used to illuminate OMCA from *trans* to *cis*. Samples were put in a 10 ml quartz crucible and stirred constantly at 25 °C in a jacketed beaker connected to a low-temperature thermostat bath. The distance between the samples and the light source was fixed at 8 cm.

### 2.5 UV-vis spectroscopy

UV-vis spectroscopy of the samples was carried out using a Shimadzu UV 2450 (Japan) spectrophotometer at 25 °C. The slit width was 2 nm, and the wavelength scanning range was from 220 nm to 360 nm. Ultrapure water was used as a blank in the experiment.

### 2.6 Rheological measurements

Rheological properties were investigated using the double gap measurement system (C-DG26.7/T200, 23.831 mm ID, 27.597 mm diameter) of the Physical MCR 302 rheometer produced by Austrian Anton Paar Co., Ltd. A sample of about 8 ml was poured into the double gap and kept stable for at least 5 min before measurements. Steady shear rheology was measured by the shear rates changing from 0.01 s^−1^ to 1000 s^−1^. The dynamic shear rheology was examined in the range of 0.05–100 Hz, with a fixed strain value (*γ* = 1%). The experimental temperature was controlled at 25.0 ± 0.1 °C.

### 2.7 Microstructure determination

The microstructures of the aggregates were obtained by a transmission electron microscope (Jeol JEM-1400, Japan). Samples for electron microscopy were prepared by negative staining (with phosphotungstate). The sample preparation process was as follows: at first, an appropriate solution was dropped onto the side of the copper mesh containing the carbon support film and left for tens of seconds, such that the samples adsorbed onto the copper mesh. The excess solution was removed with filter paper. The samples were stained with phosphotungstate for 30–50 s, and then, the excess solution was removed with filter paper for observation.

## 3 Results and discussions

### 3.1 Phase diagram of the 12-2-12·2Br^−^/*trans*-OMCA mixed system

**Fig. 1:**
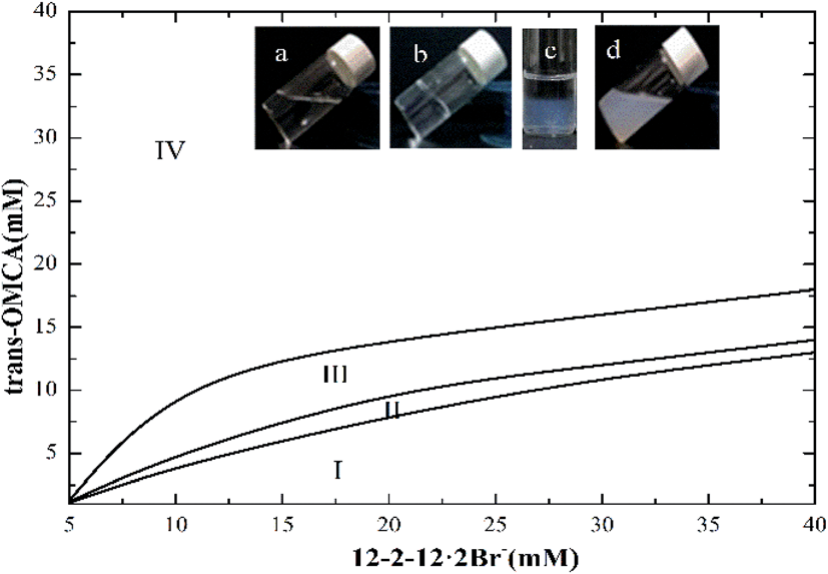
The phase diagram of the 12-2-12·2Br^−^/*trans*-OMCA mixed system at 25 °C.

The phase behaviors of the mixed solution of 12-2-12·2Br^−^/*trans*-OMCA system are complicated even if the surfactant concentration is low. Fig. 1 presents the phase diagram of the gemini surfactant 12-2-12·2Br^−^ mixed with *trans*-OMCA in a concentration ranging from 5 mM to 40 mM at 25 °C. Negative staining methods, combined with TEM observation and rheological methods, were used to examine the microstructures of aggregates in different regions. The phase diagram is divided into four regions: transparent micelle phase (region I), ATPS (Aqueous two-phase system, region II), vesicle area (region III), and solid–liquid mixed phase (region IV, precipitates). During the experiments, we found that the viscosities of the samples in the colorless and transparent region I were similar to that of pure water when the *trans*-OMCA concentration in the mixed system was low (sample a). However, with the addition of *trans*-OMCA, the viscosities and/or appearances of the samples change gradually. The significant variation is that the viscosity of the transparent micelle solution increases dramatically with an increase in the *trans*-OMCA concentration when the concentration of 12-2-12·2Br^−^ is constant (sample b). With a further increase in *trans*-OMCA concentration, the coexisting aqueous two-phase system appears (sample c) followed by a lower viscosity solution with a bluish appearance (sample d), and finally, a turbid solution is obtained. The microstructures of aggregates in the transparent micellar solution with higher viscosity and the bluish solution with lower viscosity are proved to be worm-like micelles and vesicles, respectively, by rheological measurement and/or TEM observation (see Section 3.2). In this study, the worm-like micelle system and vesicle system have been specially singled out for the study of the photoresponsive behavior because they both have more extensive applications than the others in practice.

### 3.2 Rheological properties and photoresponsive behaviors of worm-like micelle system

#### 3.2.1 Rheological properties

**Fig. 2:**
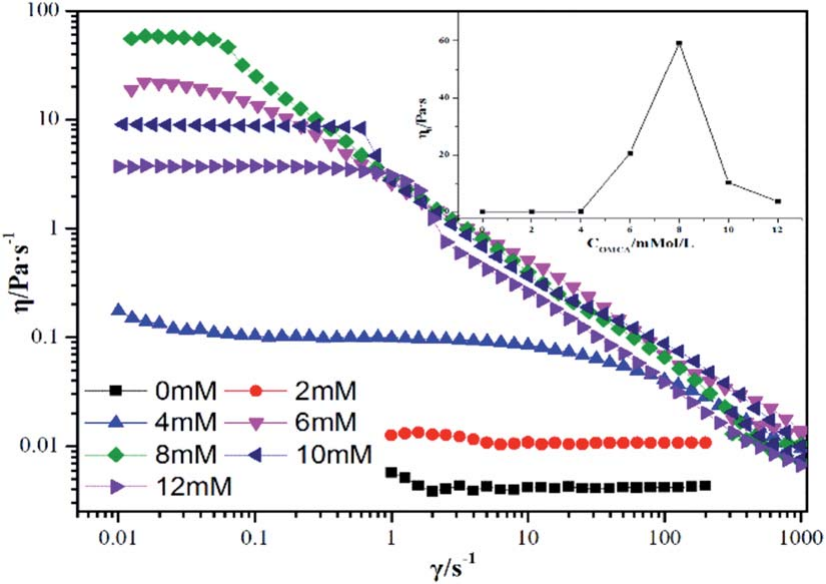
Steady-shear viscosity curves for 30 mM 12-2-12·2Br^−^ with different *trans*-OMCA (0 mM-12 mM) concentrations at 25 °C. The inset depicts the variation of zero-shear viscosity of samples with *trans*-OMCA concentration.

Worm-like micelles have extensive applications due to the special rheological properties of their aqueous solutions. Reasonably, the rheological measurements become one of the important means of determining the existence of worm-like micelles. Fig. 2 provides the steady-state viscosity curves for the mixed systems of 12-2-12·2Br^−^/*trans*-OMCA (the concentration of 12-2-12·2Br^−^ is selected as 30 mM as an example, and the concentration of *trans*-OMCA is variable). Just as mentioned in Section 3.1, when the concentration of *trans*-OMCA in the mixed system is low, the viscosity of the system is low and almost remains constant with an increase in shear rate; this system is manifested as a typical Newtonian fluid,^54^ indicating that the aggregates in the system are almost spherical micelles under this condition. However, when the *trans*-OMCA concentration is higher, the obvious shear-thinning phenomenon is observed, unambiguously implying the presence of worm-like micelles. The worm-like micelles will further entangle with each other and tend to form a three-dimensional network structure. Correspondingly, the viscosity of the system increases with an increase in the *trans*-OMCA concentration, as shown in Fig. 2. The formed network structure is difficult to be destroyed under relatively lower shear rate; therefore, the platform appears. The viscosity at the platform is generally defined as zero shear viscosity (*η*_0_).^55^ The shear-thinning phenomenon and *η*_0_ are the typical sign of worm-like micelles,^56^ which usually can be observed in mixed systems of certain surfactants and salts.^57^ The inset in Fig. 2 depicts the variation of *η*_0_ of 30 mM 12-2-12·2Br^−^/*trans*-OMCA system. It can be seen that the *η*_0_ value increases with an increase in *trans*-OMCA concentration and is up to the maximum of 59.8 Pa s when the concentration of *trans*-OMCA is increased to 8 mM.

**Fig. 3:**
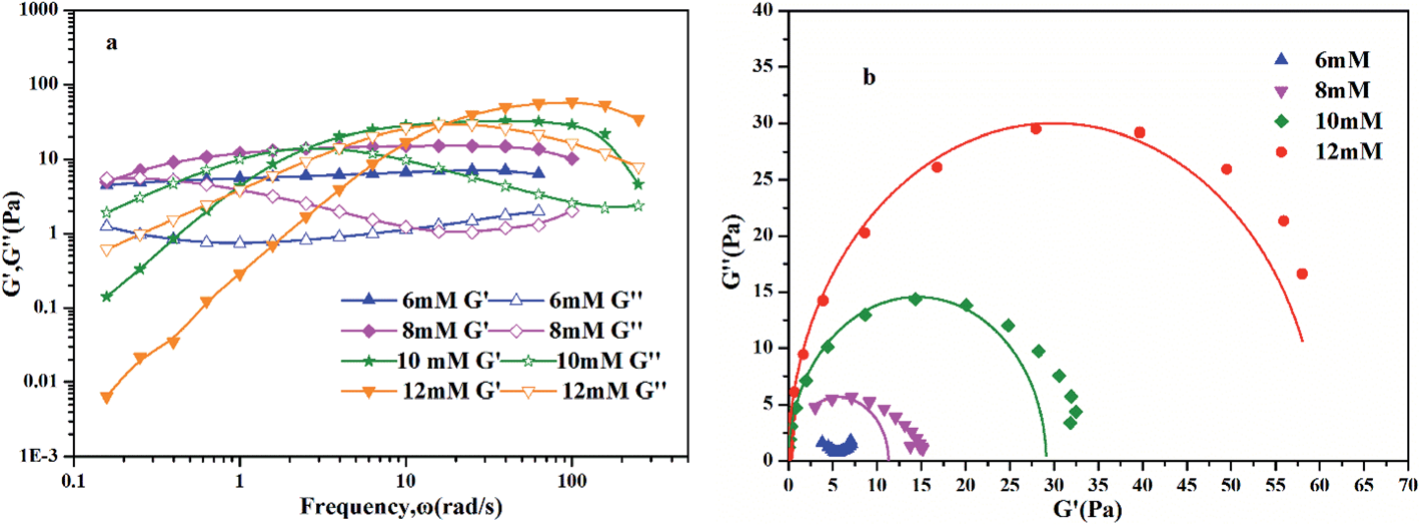
Dynamic frequency sweep (a) and Cole–Cole plots (b) for 30 mM l^−1^ 12-2-12·2Br^−^with different concentrations of *trans*-OMCA (6 mM, 8 mM, 10 mM, and 12 mM) at 25 °C (*G*′: elastic modulus; *G*′′: viscous modulus).

To further understand the viscoelastic properties of the present systems, plots of the viscoelastic modulus (*G*′: elastic modulus; *G*′′: viscous modulus) *versus* the frequencies are provided in Fig. 3. As can be seen from Fig. 3a, when the concentration of OMCA is lower than 6 mM, the system does not comply with the typical Maxwell model.^58^ When the concentration of *trans*-OMCA is higher than 8 mM, the system shows a viscous behavior of *G*′′ > *G*′ at low-frequency regions; then, both the *G*′ and *G*′′ increase and cross at a specific frequency. At higher frequencies, *G*′ and *G*′′ continue to grow and *G*′ surpasses *G*′′ (*G*′′ < *G*′), and eventually the gradient of *G*′ tends to be gentle. This phenomenon conforms to the typical Maxwell's model, indicating the formation of worm-like micelles. The related parameters can be expressed by the following formulas:^59^1
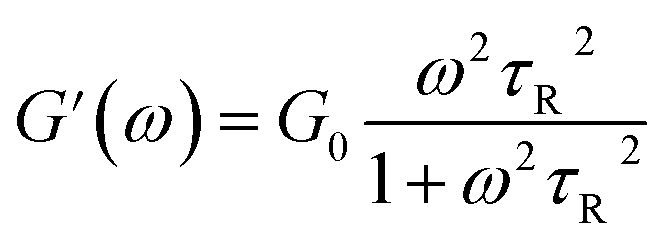
2
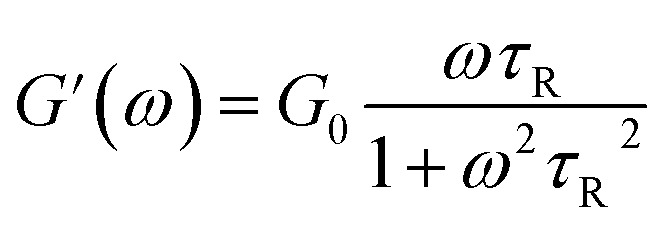
3
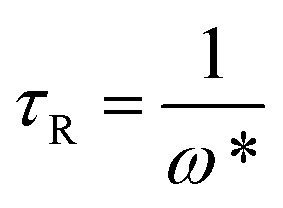

4 *G*_0_ = 2G*

herein, *G*_0_, *ω*, and *τ*_R_ are the plateau modulus, oscillation frequency, and relaxation time, respectively. ω* and G* are the angular frequency and modulus when *G*′ intersects with *G*′′. The *G*_0_ can be estimated from eqn (4) when *G*_0_ cannot be obtained by *G*′ for some experiments. The Cole–Cole plots can also be used to verify whether these rheological data fit with the Maxwell model. It can be described by the following equation:^60^5
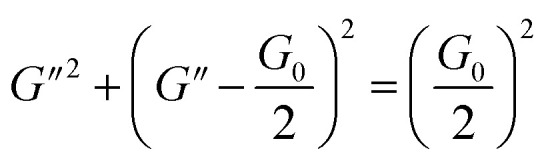


Data points of the viscous modulus *G*′′ *versus* the elastic modulus *G*′ for different samples are exhibited in Fig. 3b. Solid curves were calculated and fitted according to eqn (5). Obviously, the data perfectly follows a semicircle at low frequencies, proving the formation of viscoelastic worm-like micelles in solution. The results slightly deviate from the semicircle Cole–Cole plots at higher frequencies; this phenomenon is usually explained by Rouse modes.^61^

#### 3.2.2 Photoresponsive behaviors

**Fig. 4:**
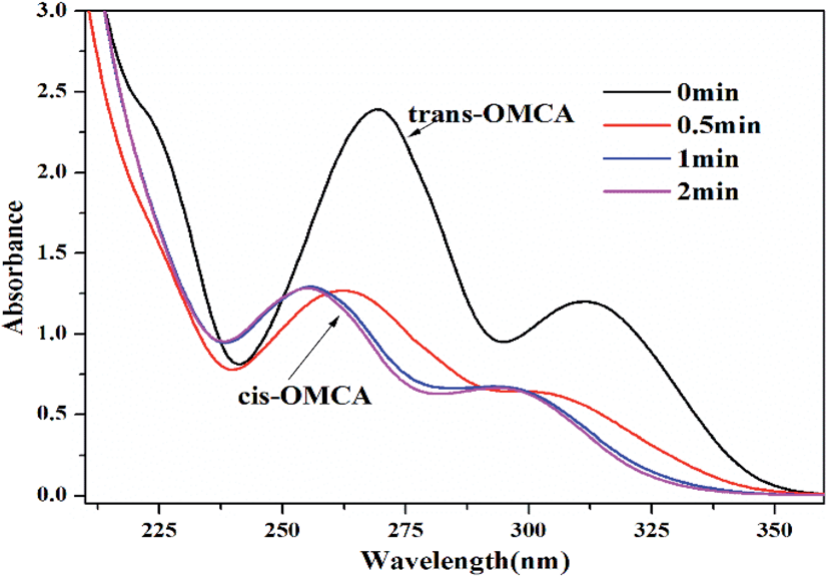
UV-vis absorption spectra of 0.1 mM *trans*-OMCA solution under different UV irradiation time at 25 °C.

The photoisomerization of the selected photosensitive molecules *trans*-OMCA is studied initially by measuring the UV-vis spectrum before and after UV irradiation, as shown in Fig. 4. The typical absorption peaks at 270 nm and 312 nm for *trans*-OMCA are clearly present in Fig. 4. The UV light irradiation results in the sample absorbance peaks shifting gradually to about 254 nm and 293 nm, which are assigned to the characteristic absorbance peaks of *cis*-OMCA.^42^ Moreover, the height of the absorbance peaks reduces significantly after UV irradiation. All these results prove that *trans*-OMCA is gradually isomerized to *cis*-OMCA under UV irradiation. Furthermore, under the studied conditions, it can be seen that *trans*-OMCA can photoisomerize to *cis*-OMCA completely within 2 min; this indicates that *trans*-OMCA has a sensitive and efficient light response.

**Fig. 5:**
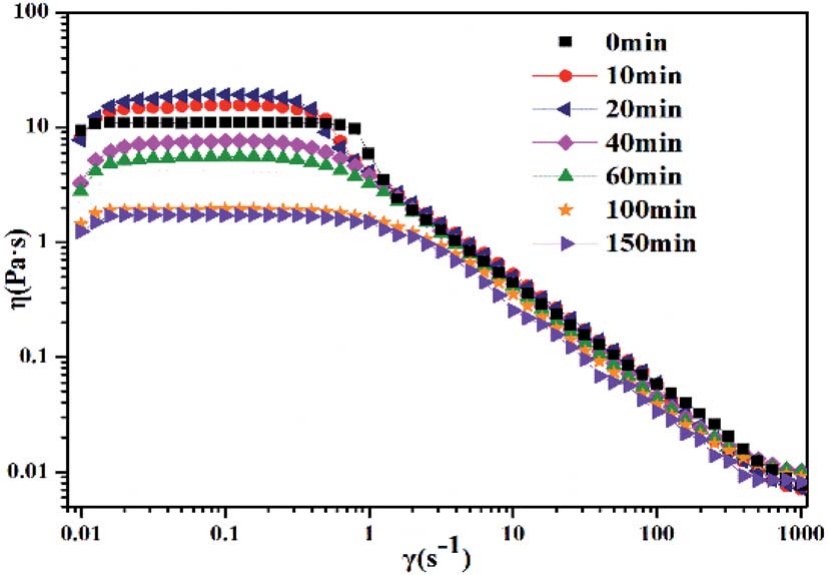
Effect of UV irradiation time (0 min, 10 min, 20 min, 40 min, 60 min, 100 min, and 150 min) on steady shear viscosity for 30 mM 12-2-12·2Br^−^/10 mM *trans*-OMCA system at 25 °C.

The photoinduced transitions in morphology of the worm-like micelles (take 30 mM 12-2-12·2Br^−^/10 mM *trans*-OMCA as an example) were explored by measuring the rheological behavior. Fig. 5 demonstrates the effect of UV irradiation time on the steady shear viscosity of 30 mM 12-2-12·2Br^−^/10 mM *trans*-OMCA system. As can be seen, when the UV irradiation is exposed to the system, the trend of the steady shear curve shows no obvious changes, and the system maintains shear dilution behavior; this indicates that the aggregates in this system are still worm-like micelles after UV irradiation. However, the zero shear viscosity of the system varies with illumination time. With the increase of irradiation time, *trans*-OMCA molecules are gradually photoisomerized to *cis*-OMCA. A previous study has proved that the *cis*-OMCA molecule has stronger hydrophilicity as compared to *trans*-OMCA.^62^ Therefore, the benzene ring molecule moves toward the outside of the micelles due to the change in configuration; this results in cation-π interaction between the OMCA molecule and the surfactant head group.^63^ Molecules are more closely integrated to promote the growth of worm-like micelles, along with a viscosity increase. With a further increase in the irradiation time (longer than 20 min), more and more *trans*-OMCA molecules are photoisomerized to *cis*-OMCA. The stronger hydrophilicity and steric hindrance effect of *cis*-OMCA molecules decrease the interactions between *cis*-OMCA and 12-2-12·2Br^−^ to some degree and thus endow *cis*-OMCA with the opportunity to move away from the micelles and enter the bulk phase; this results in the increase in the electrostatic repulsion between the surfactant head groups. Correspondingly, the worm-like micelles become shorter and shorter, leading to a decrease in the degree of entanglement of the worm-like micelles, and the viscosity of the solution decreases. The results in Fig. 5 have shown that the system is in equilibrium after exposure to UV light for 100 min, and the viscosity of the system reduces by about one order of magnitude.

**Fig. 6:**
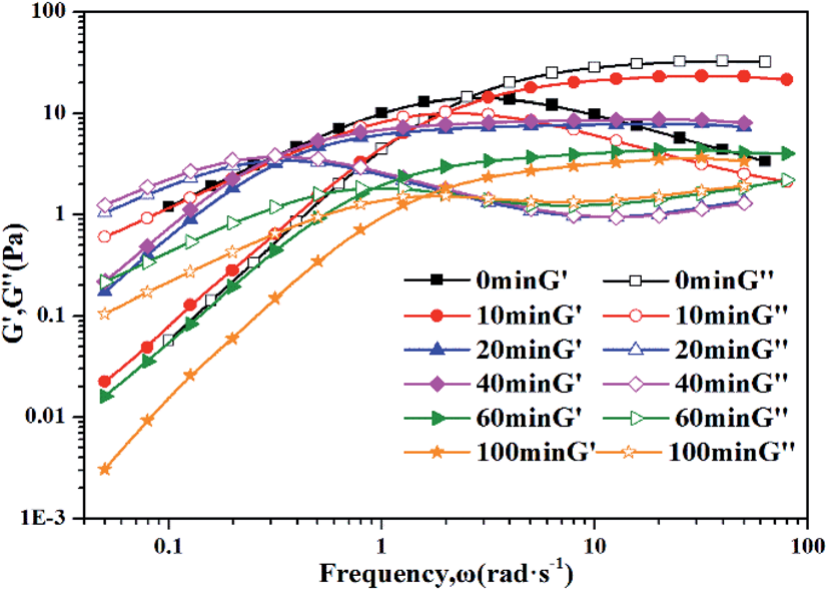
Dynamic frequency sweep of 30 mM 12-2-12·2Br^−^/10 mM *trans*-OMCA with different UV irradiation time at 25 °C.

Fig. 6 shows the dynamic frequency sweep of 30 mM 12-2-12/10 mM *trans*-OMCA with different UV irradiation times. It can be seen that the system displays the behavior of typical worm-like micelles both before and after illumination. Obviously, the self-assembled form of micelles shows no obvious changes. The average elongation length of worm-like micelles is the reflection of the rheological behavior of the system and can be calculated according to the following formula:^64^6
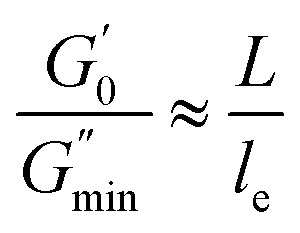
where *L* is the average stretching length and *l*_e_ is the equivalent length between two entangled points of the worm-like micelles. For worm-like micelles, *l*_e_ is generally taken 80–150 nm to calculate the value of *L*.^64^
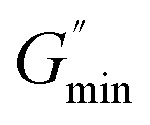
 is the minimum value of the energy consumption modulus at high frequency, 
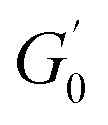
 is the platform value of the storage modulus. According to the formula (6), the rheological parameters of worm-like micelles before and after irradiation were calculated, as shown in Table 1. It can be seen from Table 1 that the average stretching length *L* of worm-like micelles slightly increases at about 10 minutes irradiation. With the prolongation of irradiation time, *L* decreases gradually. The 
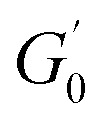
 decreases with the irradiation time. Generally, 
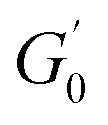
 is related to the number density of micellar aggregates; thus, the shortening of worm-like micelles will result in a decrease in the degree of entanglement between micelles and a decrease in the number density of aggregates. Obviously, the UV irradiation can induce worm-like micelles to become shorter and tend to convert into rod-like micelles. The results coincide with that obtained by steady shear measurements, as shown in Fig. 2.

**Table 1:** Rheological parameters of 12-2-12·2Br^−^/*trans*-OMCA (30 mM : 10 mM) under different UV-light irradiation time at 25 °C

System	UV light	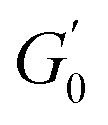 (Pa)	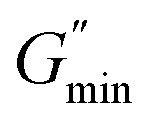 (Pa)	*L*
12-2-12·2Br^−^/*trans*-OMCA (30 mM : 10 mM)	0 min	32.660	3.300	791–1484
	10 min	21.480	2.000	859–1611
	20 min	8.320	0.930	715–1341
	40 min	7.890	0.960	657–1233
	60 min	4.250	1.195	284–533
	100 min	3.490	1.379	202–379

The abovementioned analysis has shown that the morphologies of worm-like micelles formed in the 12-2-12·2Br^−^/OMCA system are determined by the form of OMCA, and the morphologies of micelles in the 12-2-12·2Br^−^/*trans*-OMCA mixed system, can be tailored by adjusting the duration of UV light irradiation. Correspondingly, the rheological behavior of the 12-2-12·2Br^−^/OMCA mixed system can be purposefully tuned.

### 3.3 Photoresponsive behaviors of the vesicle system

**Fig. 7:**
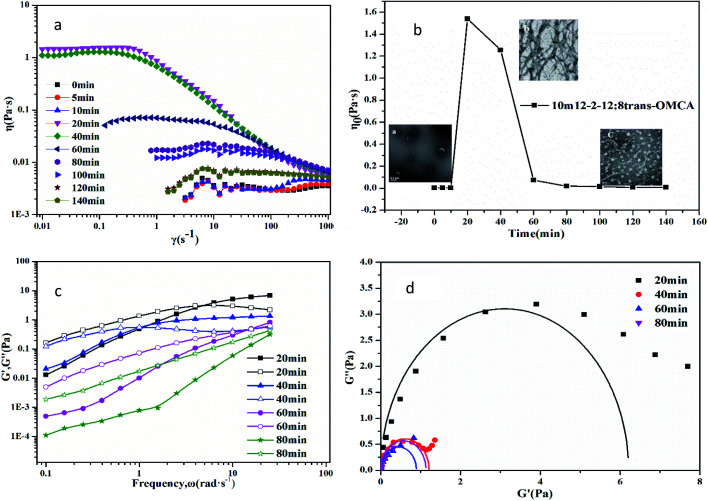
Photoresponsive viscoelasticity of 10 mM12-2-12·2Br^−^/8 mM *trans*-OMCA system at 25 °C; (a) steady shear viscosity, (b) zero-shear viscosity, (c) dynamic frequency sweep, and (d) Cole–Cole plots.

The microstructures of the samples that are bluish in appearance (region III of Fig. 1) are characterized by a negative staining method combined with TEM observation. The typical bilayer structures, namely vesicles, are observed, as shown in the inset in Fig. 7b. Fig. 7a shows that the viscosity of the vesicle solution (take 10 mM 12-2-12·2Br^−^/8 mM *trans*-OMCA as an example) is low, and the solution behaves as a typical Newtonian fluid. However, the obvious shear-thinning phenomenon appears when the sample is exposed to UV irradiation for 20 min, and the viscosity of the solution increases by three orders of magnitude; this indicates that the vesicles transform into worm-like micelles under UV irradiation. The TEM image in Fig. 7b can also prove the formation of worm-like micelles. The viscoelasticity curves in Fig. 7c and Cole–Cole plots in Fig. 7d affirm the formation of worm-like micelles. Interestingly, the viscosity of the system will decrease gradually if the UV irradiation time is prolonged over 20 min. When the irradiation time is longer than 120 min, the solution will behave as a Newtonian fluid again. The TEM image (inset in Fig. 7b) shows that the microstructure of aggregates in mixed systems is not original vesicles but spherical micelles with a diameter of about 8 nm. Obviously, the studied self-assembled system can realize a three-state transition (vesicles, worm-like micelles, and spherical micelles) by controlling the UV irradiation time. The change in the FTIR spectrum (Fig. 1s and 2s†) also indicates that the added *trans*-OMCA participates in the formation of self-assembles, and the photo isomerization of *trans* to *cis* induced by the UV light leads to a change in the self-assembly structure of aggregates.

### 3.4 Mechanism of morphological transformation of micelles induced by UV irradiation

The morphologies of micelles depend on many factors including the properties of the species that the formed micelles, the properties of solvents, as well as circumstance factors (*i.e.* the conditions under which the micelles are formed). The critical accumulation parameter (CPP) is the most important parameter to predict the self-assembly behavior of amphipathic molecules in micelles, proposed by Israelachvili and his colleagues.^65^ CPP can be expressed by the following equation:7
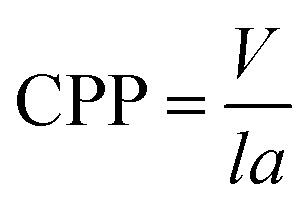
where *V* is the hydrophobic basis volume, *l* is the length of the hydrophobic chain, and *a* is the effective cross-sectional area, where the hydrophilic head group is tightly packed into a single layer. Generally, the CPP meet the following rules: when CPP ≤ 1/3, spherical micelles are usually formed; when 1/3 < CPP ≤ 1/2, the formation of rod-shaped or worm-like micelles is benefitted; when 1/2 < CPP ≤ 1, the species is apt to self-assemble into a bubble structure; when CPP = 1, the formation of planar bimolecular layers is favored; and if CPP > 1, reverse micelles usually appear. For the present system, the introduction of the sodium salt of cinnamate decreases the electrostatic repulsion between the gemini surfactant head groups and compresses the double layer by participating in the formation of mixed micelles due to the hydrophobic effect and electrostatic interactions between the gemini surfactant and OMCA. Correspondingly, *a* decreases and the CPP increases (1/3 < CPP ≤ 1/2), leading to the spherical micelle transformation into the longer worm-like micelles. If the *trans*-OMCA concentration is increased further, the micellar surface double layer becomes more compressed, and the linear worm-like micelles will self-curl into global vesicles with the CPP increased to 1/2–1.

**Fig. 8:**
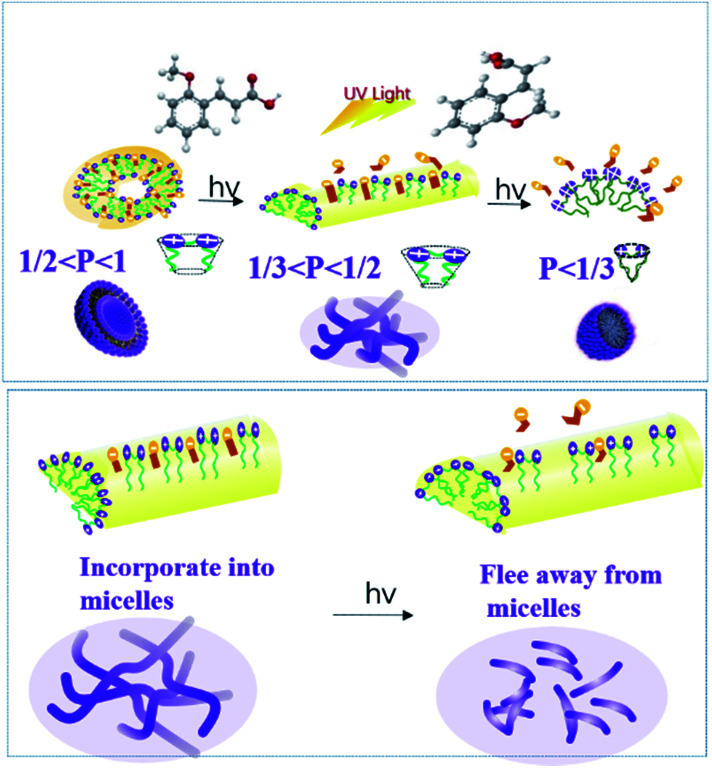
Mechanism of the aggregation behavior for the 12-2-12·2Br^−^/*trans*-OMCA system before and after UV irradiation.

The hydrophobic effect and electrostatic interactions between the gemini surfactant and OMCA are the foundation of forming aggregates with different morphologies. The results have shown that the worm-like micelles and vesicles in the present study will transform into other morphologies under UV irradiation. It is evident that the isomerization of *trans*-OMCA to *cis*-OMCA plays a decisive role in the morphological transition. The hydrophobicity of *trans*-OMCA is stronger than that of *cis*-OMCA, which provides *cis*-OMCA molecules with the chance to move towards the outer layer of micelles and even escape from the originally mixed micelles with the synergistic action of steric hindrance. Consequently, the net charges carried by micelles increase, and the effective cross-sectional area increases. Correspondingly, the flexible long worm-like micelles break into shorter rod-like micelles, and the vesicles transform into worm-like micelles and further spherical micelles with the increase in the degree of photo isomerization. The slight growth of worm-like micelles within 10 min can be explained by the fact that the benzene ring of the OMCA molecule moves upward due to a change in molecular configuration and produces a cation-π interaction; this results in a closer combination between the 12-2-12·2Br^−^ and *trans*-OMCA and promotes the growth of worm-like micelles. Fig. 8 illustrates the possible mechanism of morphological transformation of the micelles induced by UV irradiation. In a word, the differences in hydrophobicity and the steric hindrance effect between *trans*-OMCA and *cis*-OMCA provide the feasibility of creating a novel system with abundant self-assembly morphology and rheological behaviors tunable by photoinduction.

## 4 Conclusions

We constructed a simple photoinduced self-assembly system by introducing the photoresponsive derivative *trans*-OMCA into the gemini surfactant 12-2-12·2Br^−^ aqueous solution. The mixed system of 12-2-12·2Br^−^/*trans*-OMCA displays abundant phase behaviors even at lower surfactant concentrations. The photoisomerization of OMCA from the *trans* form to the *cis* form affects the degree of OMCA participation in the formation of mixed micelles, alters the molecular packing at the micellar interface, and ultimately leads to the transformation of micellar morphologies. The longer worm-like micelles turn into much shorter units, accompanied by the decrease of solution viscosity by more than an order of magnitude. The vesicles formed, however, can be utilized to generate multi-state self-assembly structures, including worm-like micelles and small spherical micelles, depending on the UV irradiation time. The hydrophobic effect and the electrostatic interactions between the gemini surfactant and OMCA are the foundation of aggregate formation with different morphologies. The difference in hydrophobicity and the steric hindrance effect between *trans*-OMCA and *cis*-OMCA means that it is feasible to create a novel system with abundant self-assembly morphology and rheological behaviors tunable by photoinduction. The morphologies of micelles in the 12-2-12·2Br^−^/*trans*-OMCA mixed system can be tailored by adjusting the system composition and duration of UV light irradiation. The rheological behavior of 12-2-12·2Br^−^/*trans*-OMCA system can be tuned deliberately. It should be noted that the coexisting aqueous two-phase system (region II in Fig. 1) will turn into a single phase, and a certain single phase around the aqueous two-phase system will separate into two phases under UV irradiation. This may provide a novel strategy for the separation and purification of certain substances. The corresponding study is still in progress. This light-induced system with abundant self-assembly behaviors and tunable rheological properties has wide application prospects in many fields such as in drug delivery, smart fluids, and materials science.

## Conflicts of interest

There are no conflicts to declare.

## Supplementary Material

RA-008-C8RA01070F-s001
