# Supplementary material for: A gemini surfactant-containing system with abundant self-assembly morphology and rheological behaviors tunable by photoinduction
Source: RSC Adv. 2018 Apr 30;8(29):16004–12. doi: 10.1039/c8ra01070f (PMC9080092; doi:10.1039/c8ra01070f)
Supplement: RA-008-C8RA01070F-s001 [file RA-008-C8RA01070F-s001.pdf]

Electronic Supplementary Material (ESI) for RSC Advances.
This journal is © The Royal Society of Chemistry 2018

Supporting Information

1 Experimental Method

Infrared spectroscopy Fourier transform infrared (FT-IR) spectra were recorded using the Thermo Fisher Nicolet 6700 FT-IR spectrometer. The freeze-dried samples were prepared by KBr disc or film technique and the scan range was 4000–400 cm^{-1}.

2 Results and Discussion

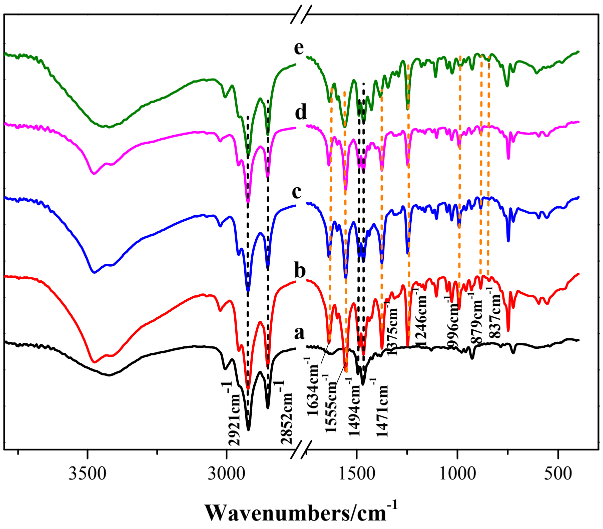

Fig. 1s FTIR spectra of 12-3-12·2Br-(a) and 12-3-12·2Br-/trans-OMCA (10mM: 8mM) sample after being irradiated with UV light for 0, 20, 60 or 100 min.

The FT-IR spectra of 12-3-12·2Br$^-$/trans-OMCA samples (a: 12-3-12·2Br$^-$; b, c, d, e: 10mM12-3-12·2Br$^-$/8mMtrans-OMCA samples before and after being irradiated with UV light (20, 40 or 60 min) were acquired. As can be seen from the Fig. 1s, the 2921 cm^{-1} and 2852 cm^{-1} are symmetrical and asymmetrical stretching vibrations of the alkyl chain C-H bond. The 1494 cm^{-1} and 1471 cm^{-1} are in-plane bending vibrations of methyl and methylene groups. Those peaks are linked to 12-3-12·2Br$^-$(a). For the 12-3-12·2Br$^-$/ *trans*-OMCA sample, the FT-IR spectrum of sample without UV light irradiation are showed in curve b. and the c, d, e represent that the sample were illuminated 20, 40, 60min respectively. All spectra present the characteristic bands of the acrylic C=C vibration at 1634 cm^{-1} and 1555cm^{-1}, and the =C-H in-plane bending modes are observed at 1374cm^{-1} and 1246cm^{-1}, indicating that the trans-OMCA participates in the self-assembly of 12-3-12·2Br$^-$ micelles. Trans and cis

isomers can be distinguished by the position of their acrylic out-of-plane =C-H bending bands located at 879cm^{-1} [1] and 837 cm^{-1} [2], respectively. Fig.1s shows that the band at 879 cm^{-1} decreases and the intensity at 837cm^{-1} increases with the prolongation of UV irradiation time. Furthermore, trans-OMCA presents a characteristic aromatic C-H in-plane bending vibration located at 996 cm^{-1}, which decreases with the UV irradiation time. This change are coherent with photoisomerization of trans to cis [3], indicating that the addition ofed trans-OMCA participate the formation of aggregates, and the photoisomerization of trans to cis induced by the UV light lead to change of self-assembly structure.

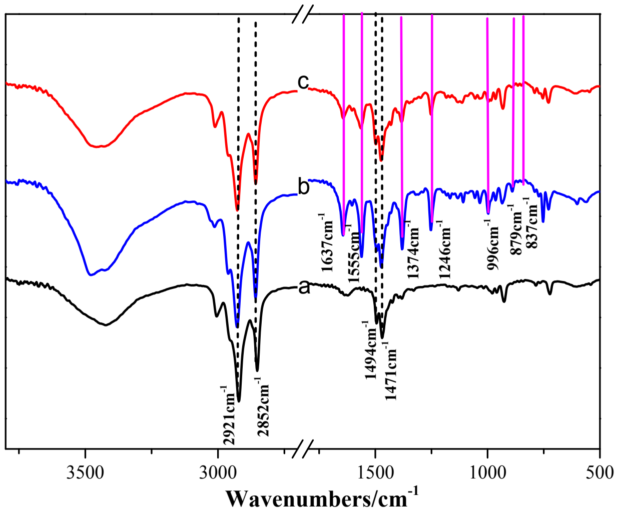

Fig. 2s FTIR spectra of 12-3-12·2Br-(a) and 12-3-12·2Br-/trans-OMCA (30mM: 10mM) sample after being irradiated with UV light for 0, 20, 60 or 100 min.

The Fig.2s shows the FT-IR spectra of wormlike micelle sample (30:10) before and after UV light. The trend of spectral variation is consistent with Fig.1s.

References:

[1]Arjunan V, Anitha R, Thenmozhi S, et al. Journal of Molecular Structure, 2016, 1113:42-54.

[2]Arjunan V, Anitha R, Marchewka M K, et al. Journal of Molecular Structure, 2015, 1080:122-136.

[3]Pallares R M, Wang Y, Lim S H, et al. Nanomedicine, 2016, 11(21):2845-2860.
